# TREM2 ameliorates neuroinflammatory response and cognitive impairment via PI3K/AKT/FoxO3a signaling pathway in Alzheimer’s disease mice

**DOI:** 10.18632/aging.104104

**Published:** 2020-10-16

**Authors:** Yaping Wang, Yan Lin, Linhan Wang, Hongrui Zhan, Xiaoting Luo, Yanyan Zeng, Wen Wu, Xingmei Zhang, Fang Wang

**Affiliations:** 1 Department of Rehabilitation, Zhujiang Hospital, Southern Medical University, Guangzhou 510282, China; 2 Key Laboratory of Mental Health of the Ministry of Education, Guangdong-Hong Kong-Macao Greater Bay Area Center for Brain Science and Brain-Inspired Intelligence, Guangdong Province Key Laboratory of Psychiatric Disorders, Department of Neurobiology, School of Basic Medical Sciences, Southern Medical University, Guangzhou 510515, China; 3 The First Affiliated Hospital, Southern Medical University, Guangzhou 510515, China; 4 Department of Radiation Oncology, Nanfang Hospital, Southern Medical University, Guangzhou 510515, China

**Keywords:** TREM2, neuroinflammation, signal transduction, microglia, Alzheimer disease

## Abstract

Triggering receptor expressed on myeloid cells 2 (TREM2) has been shown with a neuroprotective function against inflammation and neuronal injury in Alzheimer’s disease (AD). However, the TREM2 induced anti-inflammatory mechanism is still not well known. In this study it has been demonstrated that the expression of TREM2 was upregulated in hippocampus of 5xFAD mice, whereas TREM2 knock-out mediated by AAV significantly increased the levels of pro-inflammatory cytokines and aggravated cognitive defect. Additionally, FoxO3a, a downstream member of the PI3K/AKT pathway, could be activated by TREM2 defect via the PI3K/AKT signaling in 5xFAD mice. That suggests TREM2-induced protection is associated with the PI3K-FoxO3a axis. On the contrary, overexpression of TREM2 alleviated the LPS-induced inflammatory response and induced M2 phenotype microglia in vitro. This phenomenon can be abolished by applying the PI3K inhibitor LY294002, suggesting FoxO3a not only participates in TREM2-induced anti-inflammation response, but is also involved in regulating the phenotype of microglia. Taken together, our results show that the protective functions of TREM2, both in inflammatory response and cognitive impairment as well as in the decrease of M1 phenotype microglia, are related to PI3K/AKT/FoxO3a signaling pathway in AD mice.

## INTRODUCTION

Alzheimer's disease (AD), an age-dependent neurodegenerative disease, is considered the most common cause of dementia and is characterized by extracellular amyloid plaques and intracellular neurofibrillary tangles in the brain and up to now there have been no effective treatments [1, 2]. There will be 152 million people suffering from dementia worldwide by 2050 [3]. However, the pathologic mechanism of AD is not precisely clear now. Mounting evidence has shown that neuroinflammation plays a critical role in the process of AD. Microglia, an innate immune cell in the central nervous system (CNS), constantly maintains the brain homeostasis and makes a series of response when the balance of microenvironment is disrupted in many diseases [4]. Activated microglia phagocytoses abnormal substance and releases pro-inflammatory cytokines. Moreover, these cytokines would magnify inflammatory reactions and cause neuronal injury, as well as the dysfunction of learning and memory [5, 6]. Therefore, better understanding the mechanism that underlines the activation of microglia and the neuroinflammation could advance the therapeutic strategy for AD.

Triggering receptor expressed on myeloid cells 2 (TREM2) is recently considered as second genetic risk factor only followed APOE4 in AD, it is a transmembrane protein from the immunoglobulin superfamily and mainly expressed on microglia in the CNS [7, 8]. TREM2 is involved in inflammatory response, proliferation, transport, and phagocytosis in microglia [9, 10]. The potential ligands for TREM2 include bacteria, poly-anionic molecules, apolipoproteins and lipoprotein particles [11, 12]. TREM2 binds to the adaptor, DNAX-activating protein of 12kDa (DAP12), which enacts downstream cellular response or intracellular signaling pathway [13, 14]. Mounting of studies has indicated that TREM2 knockout inhibits microglia activation and increase the load of amyloid plaques in hippocampus at the late stage of AD [15]. Knocking TREM2 out aggregates retention memory defects and impairs the clearance capacity of microglia via regulating the IL-1β/IL-1RN axis at 7 months of 5xFAD mice [16]. Moreover, rs75932628, an SNP leading to an Arg to His change at amino acid 47 (R47H), impair the TREM2 downstream activation in a TREM2-dependent manner in vitro [17, 18]. Conversely, upregulation of TREM2 in AD model mouse ameliorated spatial cognitive impairment, reduced the load of amyloid plaques and proinflammatory cytokine levels by a DAP12-dependent manner during middle age [19]. Furthermore, the TREM2-TYROBP signaling pathway inhibits proinflammatory response through inhibiting the TLR pathway [13, 20]. Although studies indicate that TREM2 inhibits pro-inflammatory cytokine levels and enhances the phagocytosis of microglia in the brain, the precise mechanism remains unclear.

Forkhead transcription factor DAF-16 (FoxO3a), from mammalian forkhead box protein O family, is a conserved transcriptional factor which is crucial for life span [21]. FoxO3a activity is modulated through post-translational modifications (PTMs) by a series of kinases and followed by translocation between nucleus and cytoplasm, regulating the genes involved in metabolism, inflammatory response, autophagy, oxidative stress and cell death [21, 22]. AKT (protein kinase B) is activated by upstream of phosphatidylinositol 3-kinase (PI3K) that is recruited downstream of TREM2. FoxO3a is modulated by the PI3K-AKT pathway [23]. Moreover, phosphorylated FoxO3a (p-FoxO3a) by calorie restriction (CR) pathway alleviated the amyloid plaques and cognitive disorder in the Tg2576 mice, suggesting that inactivation of FoxO3a attenuated the pathology of AD [24]. Exposure to Aβ_1-42_ stimulates microglia activation, which relies upon unphosphorylated FoxO3a translocating into the nucleus and can be reversed by silencing FoxO3a [25]. Interestingly, recent findings suggested that KO-TREM2 in vivo and in vitro, microglia could increase autophagy via inactivating mTOR signaling pathway in AD [10]. However, autophagy inhibitor (3-methyladeninei) could block the inflammatory response in LPS-stimulated acute injury of lung. Inactivation of FoxO3a/Autophagy signaling pathway induced by punicalagin could downregulate the increase of proinflammatory cytokines induced by LPS [26, 27]. Furthermore, TREM2 can inhibit inflammatory response through alleviating oxidative stress caused by activating the PI3K/AKT pathway [28], while tempol attenuates the inflammation via PI3K/AKT/FoxO3a pathway in obstructed kidney [29].

Based on the above, this study is carried out to address whether the FoxO3a is involved in TREM2-induced anti-inflammatory response and its related signaling pathway in the 5xFAD mice model. Finally, we found that TREM2 can attenuate inflammatory response via the PI3K/AKT/FoxO3a signaling pathway and alleviate cognitive impairment in AD mice.

## RESULTS

### The expression of inflammatory cytokines was increased at the hippocampus in 5xFAD mice

**Figure 1:**
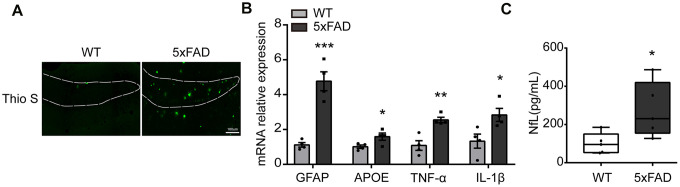
**The expression of inflammatory cytokines in the hippocampus in 5xFAD mice.** (**A**) Representative image of Aβ load stained with Thioflavine S (green) in hippocampus (DG). Scale bar, 100μm. (**B**) The expression of GFAP, APOE, TNF-α, IL-1β were evaluated by qPCR (n=4). (**C**) The quantification of serum NfL (n=5). All of the results are shown as mean ± SEM. * P<0.05, ** P<0.01, *** P<0.001, vs WT group.

To evaluate the level of inflammatory molecules in the whole hippocampus of 6-month 5xFAD mouse the amyloid plaques, associated risk factors (IL-1β, TNF-α, APOE, GFAP) and neurofilament light chain (NfL) were examined. As shown in Figure 1A, massive Aβ plaques were obviously observed in the hippocampus (DG) in 5xFAD compared to Wide Type (WT) group. qPCR results showed that the expression of apolipoprotein E (APOE), GFAP, IL-1β and TNF-α was significantly increased in 5xFAD mice (Figure 1B). The serum NfL, a marker of AD, was also prominently upregulated (Figure 1C). All the results showed that inflammatory response was increased in the hippocampus of 5xFAD mice, compared to WT mice [12, 30].

### The expression and localization of TREM2 and FoxO3a in the hippocampus in 5xFAD mice

**Figure 2:**
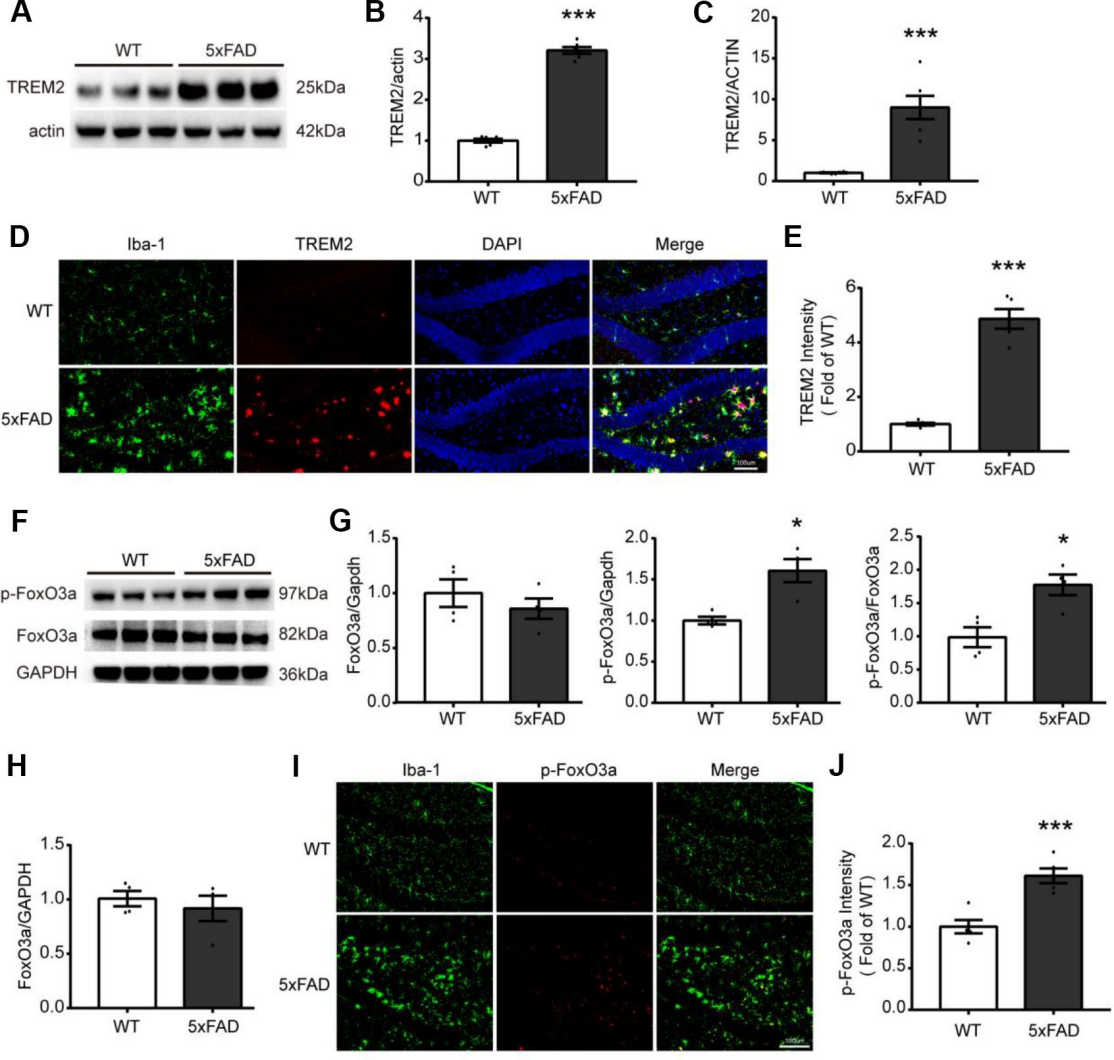
**The expression and localization of TREM2 and FoxO3a in the hippocampus in 5xFAD mice.** (**A**, **B**) Western blot bands and its quantificational analysis of TREM2 (n=6), respectively. (**F**, **G**) Western blot bands and its quantificational analysis of FoxO3a (n=4), respectively. In which, p-FoxO3a was normalized to total FoxO3a expression and total FoxO3a was normalized to GAPDH. (**C**, **H**) The mRNA levels of TREM2 (n=6) and FoxO3a (n=4) were measured by RT-PCR in the hippocampus. (**D**, **I**) Brain sections were doubly stained with Iba-1 (green) Ab for microglia and TREM2 Ab (red) or p-FoxO3a Ab (red) in the hippocampus from 5xFAD and WT mice at 7 months old. (**E**, **J**) Quantificational analysis of the expression of TREM2 and p-FoxO3a, respectively, which were matched with Fig D and Fig I (n=5), respectively. Original magnification, 20x; Scale bar=100 μm. * P<0.05, ** P<0.01, *** P<0.001, vs WT group. Bars were represented as mean ± SEM.

For investigating the relations between TREM2 and/or FoxO3a in AD pathology, first, the TREM2 level was examined by qPCR and western blot in whole hippocampus, in which the mRNA and protein level of TREM2 were significantly increased in the hippocampus in 5xFAD than WT (Figure 2A–2C). Additionally, the brain sections from WT and 5xFAD mice were also examined by confocal microscopy for colocalization of TREM2 and microglia marker Iba-1 (myeloid cell marker ionized calcium-binding adapter molecule 1). The results showed that the TREM2 was significantly upregulated and overlapped with Iba-1 surrounding the amyloid plaques, and that more amoeboid phenotype (activated) microglia in the DG area of hippocampus in 5xFAD mice (Figure 2D, 2E). These results revealed the TREM2 was mainly expressed on amoeboid microglia and was upregulated in 5xFAD mice. The mRNA and protein levels of FoxO3a were also examined (Figure 2F–2H), there is no difference in total protein or mRNA levels of FoxO3a between WT and 5xFAD mice (Figure 2G, 2H). Interestingly, the phosphorylation of FoxO3a (p-FoxO3a) was significantly increased in hippocampus in 5xFAD mice. We also examined where the p-FoxO3a locates in microglia by co-staining brain sections targeting their specific markers (p-FoxO3a and Iba-1), the results showed that more p-FoxO3a colocalized with Iba-1 in 5xFAD mice compared to WT mice (Figure 2I, 2J).

### TREM2 knockdown exacerbates cognitive and memory function in the 5xFAD mouse

**Figure 3:**
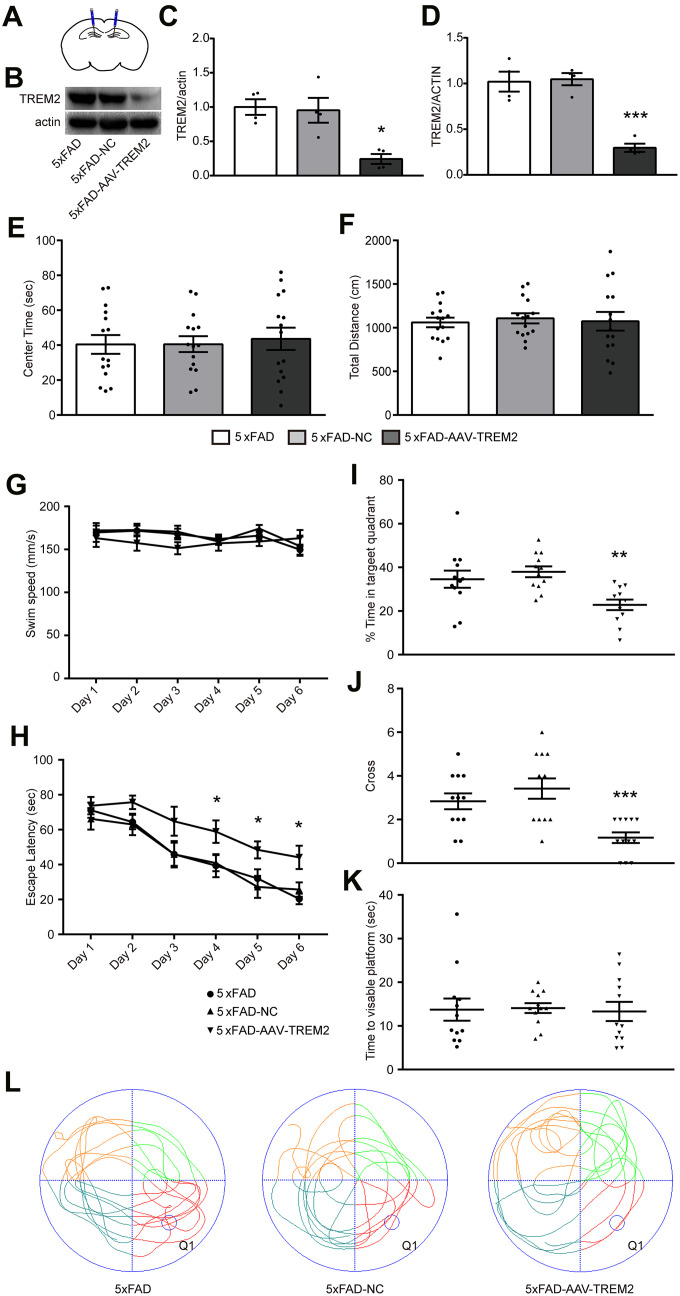
**TREM2 defect aggravated cognitive and memory function in 5xFAD mice.** (**A**) Schematic diagram of mouse brain hippocampal injection of AAV-TREM2 or control AAV. (**B**, **C**) western blot bands (**B**) and its quantificational analysis (**C**) of TREM2 in each groups (n=4). (**D**) Quantificational analysis of TREM2 mRNA by RT-PCR. (**E**, **F**) Total distance and center times were examined in open field test. (**G**, **H**) Swim speed and escape latency were recorded in training days. (**I**, **J**) Time percentage in target quadrant per group (**I**) and cross times (**J**) were recorded during the probe test. (**K**) Time to visible platform was examined in cued test. (**L**) Representative runs of three groups of mice in the probe test in MWM. Q1 was the target quadrant where the platform was located in the training days. Statistical difference was performed by two-way ANOVA and one-way ANOVA. * P<0.05, ** P<0.01, *** P<0.001, vs 5xFAD group; # P<0.05, ## P<0.01, ### P<0.001, vs 5xFAD-NC group. Results were presented as mean±SEM, n=12 per group.

To evaluate whether TREM2 affects cognitive and memory function in 5xFAD mice, all-in-one CRISPR-Cas9-AAV-TREM2 was injected into the hippocampus (DG) area of 5xFAD mice in order to downregulate TREM2 (Figure 3A). The efficiency of TREM2 knockdown was confirmed by western blot (Figure 3B, 3C) and qPCR (Figure 3D). In which the levels of TREM2 protein and mRNA were significantly declined, respectively, compared to 5xFAD-NC group, suggesting that the endogenous TREM2 gene were significantly silenced in hippocampus.

The 5xFAD, 5xFAD-AAV-NC, and 5xFAD-AAV-TREM2 groups were firstly investigated at 28 days after AAV or PBS injected to the 7 months old mice. Open-field test (OFT) was used for anxiety behavior. We found there were no significant differences among the three groups at the total distance and center time tested in the center of circular chamber for 5 min (Figure 3E, 3F). The OFT results indicated that all of the groups were in normal activity and no anxiety-like behavior.

Then, the spatial learning and memory were assessed by the Morris Water Maze test (MWM). Spatial learning was examined through continuous 6 days training trails. Swim speed in training trails was similar in all of the groups (Figure 3G). The escape latency to find the hidden platform was significantly increased on day 4 to 6 in 5xFAD-AAV-TREM2 group compared with the other two groups, and no differences between 5xFAD and 5xFAD-AAV-NC group (Figure 3H). In the probe test (hidden platform was removed away), which was used for evaluating the memory recall, the percentage of time in the target quadrant, as well as the time crossing the platform were significantly decreased in 5xFAD- AAV-TREM2 group compared to 5xFAD and 5xFAD-AAV-NC groups (Figure 3I, 3J, 3L). There were no differences in the time moving to visible platform among all groups (Figure 3K). Taken together, these results indicated that the learning and memory function was significantly attenuated in the 5xFAD-AAV-TREM2 (TREM2-lack) group without movement disorder and anxious behaviors.

### TREM2 deficiency induces FoxO3a activation and increases amyloid plaques in hippocampus in 5xFAD mice

**Figure 4:**
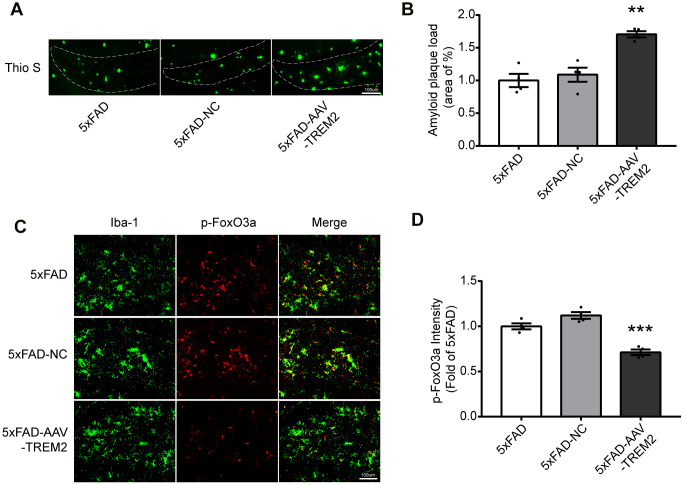
**TREM2 deficiency increases amyloid plaques and decreases p-FoxO3a level in the hippocampus in 5xFAD mice.** (**A**, **B**) Aβ load in hippocampus (DG) were stained (**A**) with Thioflavine S (green) and its quantification (**B**) in all groups (n=4 mice per group, 7 fields/mice (DG)). (**C**) Brain sections were doubly stained with Iba-1 Ab (green) and p-FoxO3a Ab (red) in the hippocampus. (**D**) Quantificational analysis of p-FoxO3a level from Fig4 C (N=4 mice per group, 9 fields/mice (DG)). Scale bar=100 μm. ***P<0.001, vs 5xFAD group; ## P<0.01, ### P<0.001, vs 5xFAD-NC group. Results were described as mean ± SEM.

First, the load of amyloid plaques was stained with Thio S to verify if TREM2 can modulate Aβ clearance of microglia. The increased Aβ was observed in 5xFAD-AAV-TREM2 group compared with other groups (Figure 4A, 4B). Next, whether TREM2 knockdown affects FoxO3a activation in microglia was investigated. The number of microglia (Iba-1) was significantly decreased around amyloid plaques in 5xFAD-AAV-TREM2 group (Figure 4C, 4D). The number of overlap cells, p-FoxO3a and Iba-1, was significantly decreased and the p-FoxO3a level was downregulated in DG area in 5xFAD-AAV-TREM2 group (Figure 4C, 4D). Taken together, TREM2 deficiency significantly activated FoxO3a (having less p-FoxO3a) and increased the amyloid plaques, suggesting FoxO3a may participate in TREM2-mediated AD pathology in the transgenic mice.

### TREM2 deficiency impairs PI3K/AKT/FoxO3a signaling and upregulates inflammatory response in vivo

**Figure 5:**
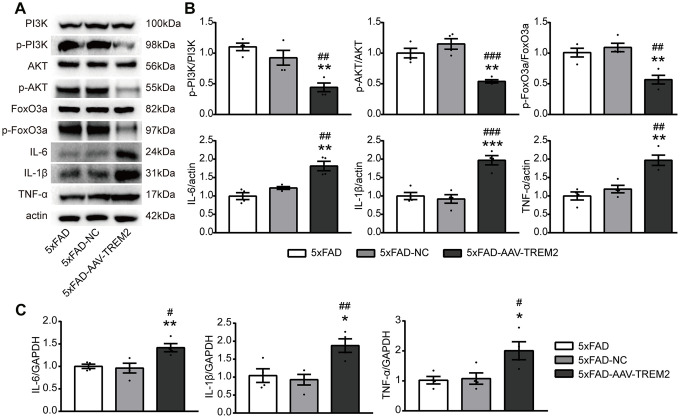
**TREM2 downregulation inactivates the PI3K/AKT/FoxO3a pathway and increases the inflammatory cytokine levels.** (**A**, **B**) Western blot bands (**A**) and quantificational analysis (**B**) of p-PI3K, p-AKT, p-FoxO3, IL-6, IL-1β and TNF-α in all groups (n=4). (**C**) The mRNA levels of proinflammatory cytokines (IL-1β, IL-6, TNF-α) were detected by qPCR (n=4 per group). *P<0.05, **P<0.01, ***P<0.001, vs 5xFAD group; # P<0.05, ## P<0.01, ### P<0.001 vs 5xFAD-NC group. Data were presented as mean ± SEM.

Several studies have demonstrated that AKT/FoxO3a pathway is involved in the inflammatory response in microglia. To further corroborate the association between TREM2 and PI3K/AKT/FoxO3a signaling, the active condition of PI3K/AKT/FoxO3a pathway and the inflammatory cytokine levels have been evaluated in the DG area of hippocampus from sacrificed mice on the 3^rd^ day after MWM by western blot and qPCR (Figure 5A, 5B). TREM2 deficiency significantly decreased the levels of downstream molecules including p-PI3K, p-AKT, p-FoxO3a and significantly increased the protein expressions of IL-6, TNF-α and IL-1β, compared to 5xFAD-AAV-NC and 5xFAD groups. Similarly, the upregulated mRNA levels of pro-inflammatory cytokine were also been observed in 5xFAD-AAV-TREM2 (Figure 5C). In summary, TREM2 deficiency inactivated the PI3K/AKT/FoxO3a pathway and increased the inflammatory cytokines in 5xFAD mice.

### Upregulation of TREM2 attenuates inflammatory response through the activation of PI3K/AKT/FoxO3a signaling in vitro

**Figure 6:**
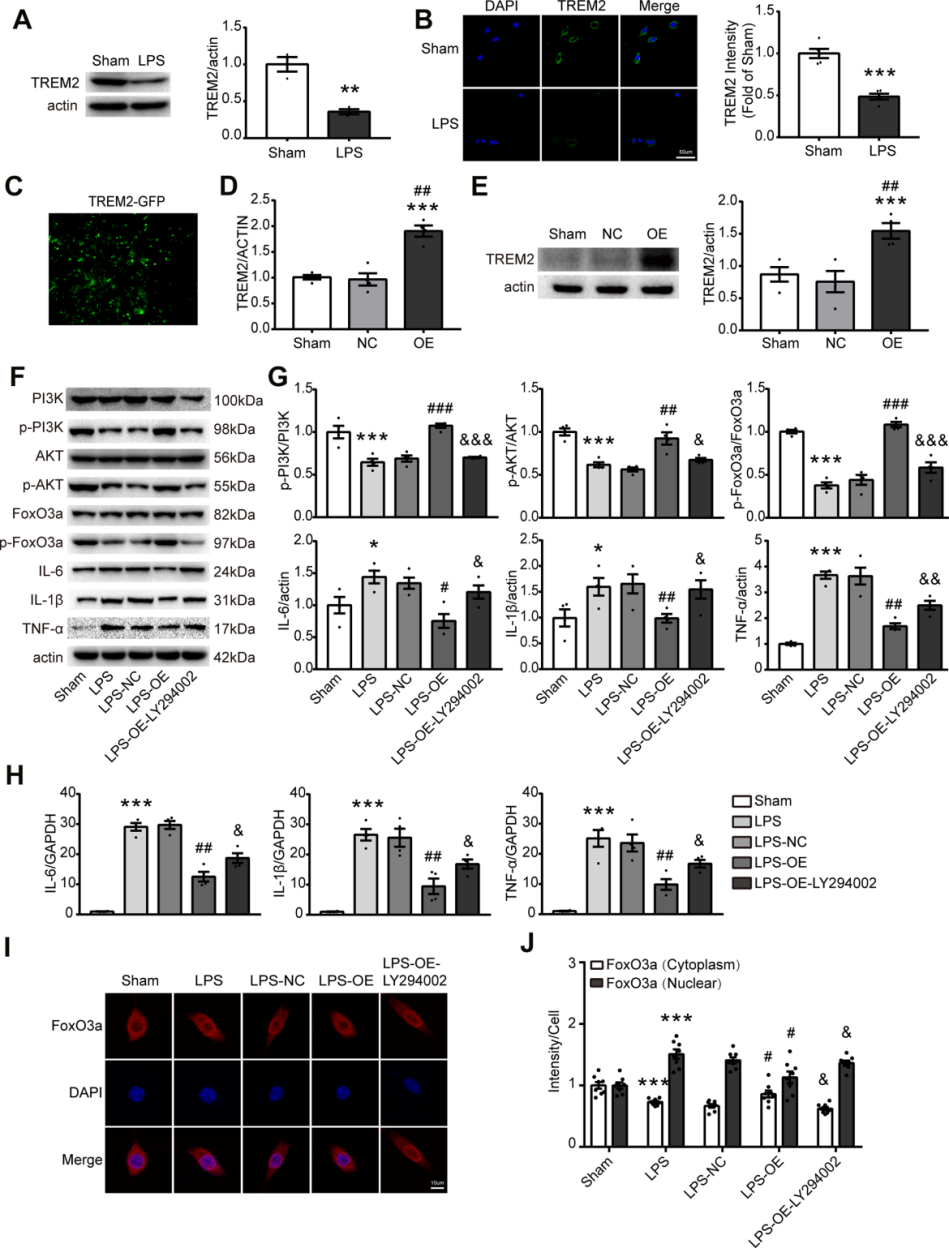
**TREM2 overexpression activates the PI3K/AKT/FoxO3a pathway and inhibits the inflammatory response in BV2 cells.** (**A**) Meaningful western blot bands and gray analysis of TREM2 (n=3), **P<0.01 vs Sham. (**B**) Confocal images and quantification analysis of fluorescence intensity of TREM2 (n=5). Scale bar, 50 μm. ***P<0.001 vs Sham. (**C**) A image of the overexpression of TREM2 plasmid after cell transfection. (**D**, **E**) TRME2 mRNA and protein were detected by qPCR and Western blot, respectively, in whole-cell lysates (n=4). ***P<0.001 vs Sham; ## P<0.01 vs NC. (**F**, **G**) Representative western blot bands (**F**) and its quantification (**G**) of p-PI3K, p-AKT, p-FoxO3, IL-6, IL-1β and TNF-α (n=4). (**H**) mRNA levels of proinflammatory cytokines (IL-1β, IL-6, TNF-α) were examined by qPCR (n=4). (**I**) The FoxO3a (red) were stained in the cells and observed by confocal microscope. (**J**) The quantification of FoxO3a in both nucleus and cytoplasm in each group (n=8); Magnification= 40x. Scale bar=10μm. * P<0.05, ** P<0.01, *** P<0.001, vs Sham; # P<0.05, ## P<0.01, ### P<0.001, vs LPS-NC; & P<0.05, && P<0.01, &&& P<0.001, vs LPS-OE. Results were represented as mean ± SEM.

To further verify that TREM2 regulates inflammatory response via the PI3K/AKT/FoxO3a signaling pathway, BV2 cells were employed for the test in vitro. Initially LPS was used to induce inflammatory response in BV2 cells, the results showed that the expression of TREM2 was decreased under LPS challenge (Figure 6A, 6B). Then, the overexpression of TREM2/plasmid (OE group) was transfected into BV2 cells. The levels of TREM2 were identified by qPCR and Western blot, the mRNA and protein levels of TREM2 were significantly increased in OE group at 72 hr after the transfection, compared with other groups (Figure 6C–6E). Upregulation of TREM2 significantly increased the phosphorylation level of p-PI3K, p-AKT and p-FoxO3a, but decreased the protein and mRNA levels of IL-6, IL-1β, and TNF-α in OE group in a TREM2-dependent manner compared to the other groups (Figure 6F–6H). The inactivation of FoxO3a means less FoxO3a remaining in the nucleus or more p-FoxO3a going into the cytoplasm from the nucleus (Figure 6I, 6J, p-FoxO3a/FoxO3a). To examine whether FoxO3a inactivation is a TREM2/AKT-dependent manner, the inhibitor of PI3K, LY294002, was used to treat the microglial cells for blocking the PI3K/AKT signaling. In this case, the protective effects of TRME2 overexpression could be reversed by LY294002. The results showed that TREM2 inhibited proinflammatory cytokine levels through PI3K-FoxO3a axis.

Our findings showed that TREM2-induced anti-inflammatory response correlated with FoxO3a inactivation and also raised the question of whether the nuclear translocation of FoxO3a is regulated by TREM2 via PI3K/AKT. To identify whether nuclear translocation of FoxO3a is mediated by TRME2, subcellular localization of FoxO3a was detected by staining total FoxO3a in the cell. The results indicated that FoxO3a translocated into the nucleus from the cytoplasm after LPS treatment, and was reversed by TREM2 upregulation. PI3K inhibitor, LY294002, abolished the effects of TREM2 overexpression (Figure 6I, 6J). Therefore the inactivation of FoxO3a attenuated LPS-induced inflammatory response and was regulated by TREM2 via PI3K/AKT pathway in microglia.

### TREM2 overexpression decrease M1 microglia phenotype via PI3K-FoxO3a axis

**Figure 7:**
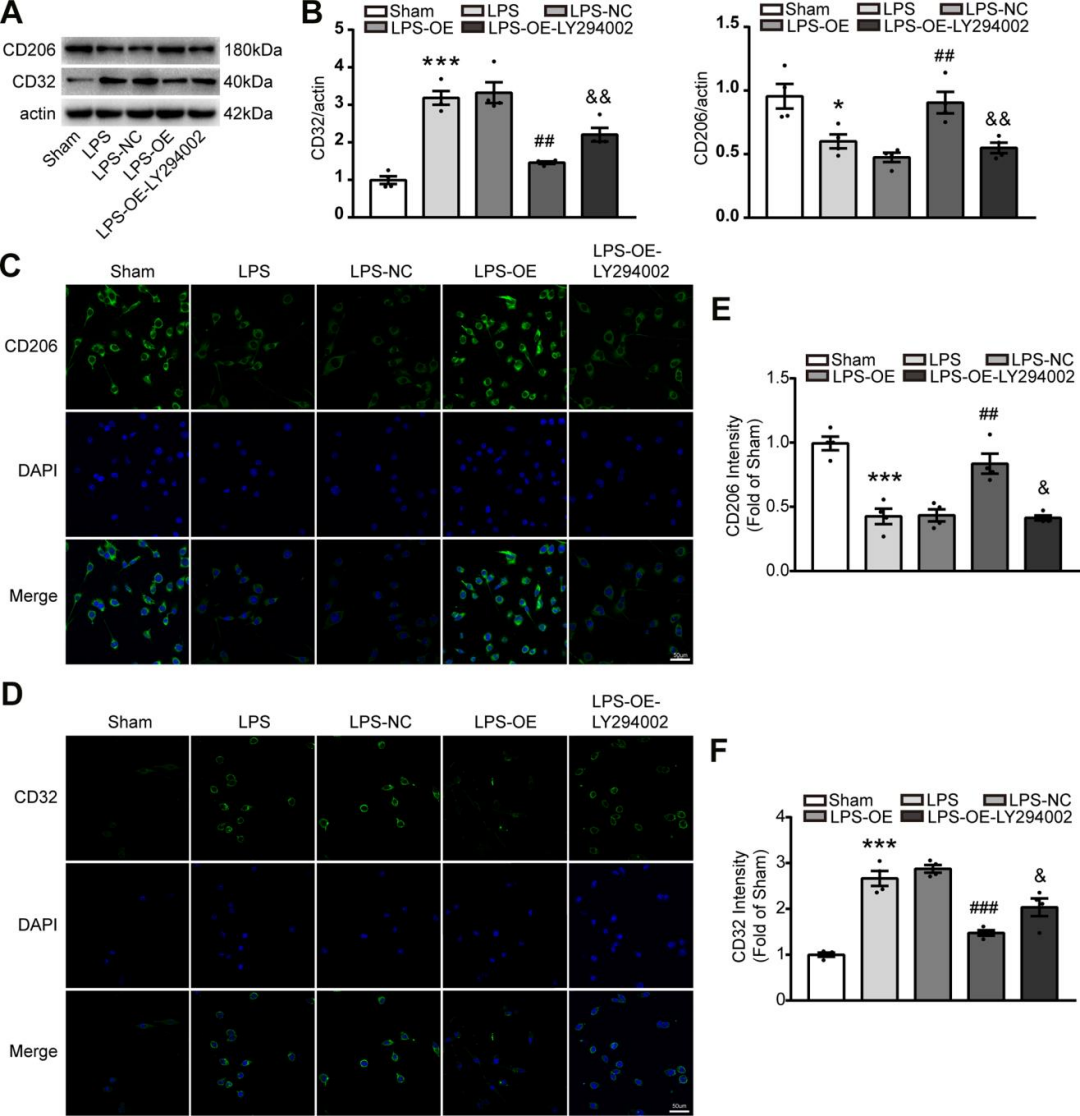
**TREM2 overexpression decreases the number of M1 microglia via PI3K-FoxO3a pathway, but increased the number of M2 microglia.** (**A**, **B**) Meaningful western blot bands (**A**) and their quantification (**B**) of CD32 and CD206 (n=4). (**C**–**F**) M2 and M1 microglia were evaluated with stained CD206 (**C**) or CD32 (**D**), respectively, by confocal microscope and their quantitative analysis of CD 206 (**E**) and CD32 (**F**). n=4 per group, 9 fields/sample. Scale bar=50 μm. * P<0.05, ** P<0.01, *** P<0.001, vs Sham; # P<0.05, ## P<0.01, ### P<0.001, vs LPS-NC; & P<0.05, && P<0.01, &&& P<0.001, vs LPS-OE. Results were showed as mean ± SEM.

Activated microglia were divided into ‘pro-inflammatory’ microglia and ‘anti-inflammatory’ microglia in the brain, they are important to inflammatory response. To investigate whether TREM2 regulated the microglia polarization via PI3K/AKT/FoxO3a axis, the protein of CD206 and CD32 were assessed by western blot, also immunocytochemistry was used to analyze the phenotypes of microglial cells after staining with anti-CD32(for M1) and anti-CD206(for M2) antibodies. The expression of CD206 significantly decreased under the stimulation of LPS, whereas TREM2 overexpression could restore it. However, the expression of CD206 induced by overexpression of TRME2 could be blocked by LY294002 (Figure 7A–7C, 7E). Conversely, the expression of CD32 markedly increased by LPS-stimulation, and could be restored by TREM2 upregulation. The expression of CD32 under TREM2 upregulation was abolished by LY294002 (Figure 7A, 7B, 7D, 7F). These data suggested that TREM2 could switch the phenotype of microglia via PI3K-FoxO3a pathway.

### Summary of the relations among TREM2 receptor, the PI3K/AKT/FoxO3a pathway and the levels of pro-inflammatory factors

**Figure 8:**
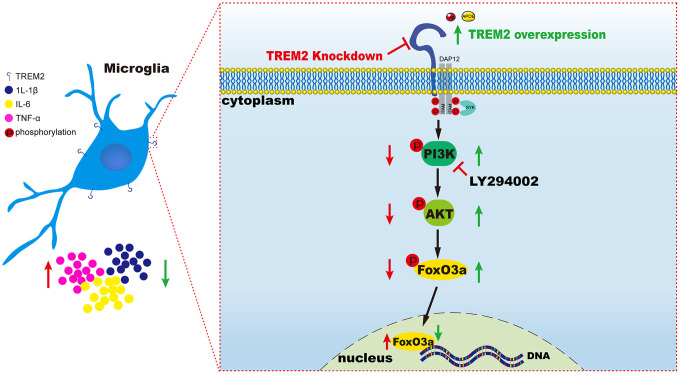
**A schematic diagram of TREM2 modulating the inflammatory response via PI3K/AKT/FoxO3a pathway in microglia.** On the left (red), the result is led from the knockdown of TREM2, while on the right (green) the result is caused by the overexpression of TREM2 receptor.

The results from our research has indicated knockdown of TREM2 could lead to the increase of pro-inflammatory cytokines mediated by the inactivation of PI3K/AKT/FoxO3a signaling pathway, conversely overexpression of the receptor would exhibit the opposite consequence through the activation of the same pathway (Figure 8).

## DISCUSSION

In this study, we explored the anti-inflammatory effects and the possible signal transduction pathway mediated by TREM2 receptor in 5xFAD mice and microglia cells. Our results showed that (1) The expression of TREM2 was significantly increased in hippocampus in 6-7 months old 5xFAD mice. The total FoxO3a protein was no difference, but the phospho-FoxO3a (p-FoxO3a) was increased in 5xFAD mice and colocalized with microglia, suggesting that Foxo3a may be involved in AD progress; (2) Downregulation of TREM2 by injecting CRISPR/Cas9-AAV-TREM2 aggregated the cognitive function and increased the amyloid plaque deposition in 5xFAD mice; (3) the PI3K/AKT/FoxO3a pathway was found to be involved in the anti-inflammatory process modulated by TREM2 in these aspects: (i) knockdown of TREM2 significantly inhibited the PI3K/AKT pathway and activated FoxO3a (that means less p-FoxO3a/FoxO3a ratio), so the inflammatory response was enhanced in 5xFAD mice. (ii) Conversely, upregulation of TREM2 activated the PI3K/AKT/FoxO3a pathway leading to the translocation of FoxO3a from nucleus to cytoplasm as p-FoxO3a form, so the proinflammatory cytokine levels were decreased in BV2 cells; (4) TREM2 could modulate the number of M1 microglia via PI3K-FoxO3a axis. Based on these data, the conclusion could be drawn that TREM2 might serve as a critical role in regulating microglial function and anti-inflammation via the PI3K/AKT/FoxO3a signaling pathway, which was one of the signal pathways involved in producing these functions.

Neuroinflammation was known as a strong propeller in AD. Mounting evidence indicated that activated microglia can release proinflammatory cytokines (IL-1β, IL-6, and TNF-α) and injure neurons, they are responsible for AD progression. Researches have demonstrated that Toll-like receptors, as well as NOD-like receptors, responded to Aβ stimulation and mediated inflammatory response in microglia [31, 32]. To maintain the brain balance, these receptors activate downstream molecules (such as nuclear factor NFκB) to translocate into the nucleus to regulate the expressions of proinflammatory cytokine in microglia [33, 34]. TREM2, an immune receptor mainly expressed on microglia in CNS, mediated proliferation, growth, phagocytosis, survival, and inflammation. Recently, deficiency of TREM2 downregulated plaque-associated microglia and activated inflammatory response in AD mouse, suggesting that TREM2 is necessary for microglia to response to amyloid plaques(Aβ) and anti-inflammation [16, 35]. The mechanism of TREM2-induced anti-inflammation in AD is still unknown. In our research, TREM2 were significantly increased and colocalized with microglia in the hippocampus in 5xFAD mice, this are in line with the previous studies that TREM2 has been increased in AD mouse models and AD patients [36, 37]. Based on our results, TREM2 do participate in the inflammatory process in AD.

Increasing evidences showed that TREM2 exhibited some protective effects, including anti-inflammation, autophagy inhibition and enhanced phagocytosis in neurodegenerative diseases, such as AD, Parkinson's disease (PD), and epilepsy [38]. Previous research demonstrated that TREM2 could ameliorate learning and memory defects through decreasing Aβ accumulation at hippocampus in AD mice [39]. Upregulation of TREM2 mitigates cognitive decline by suppressing inflammatory response via PI3K/AKT pathway in epilepsy [28]. TREM2-knockout in microglia significantly aggravated cognitive function and downregulated inflammatory genes in 5xFAD mice [16]. Moreover, the decrease of microgliosis was observed in TREM2 mutant R47H patients [13]. Consistent with the previous studies, in this study, knockdown of TREM2 aggravated learning and memory disorder without affecting anxious behavior and significantly increased the level of pro-inflammatory cytokines in 5xFAD mice. Conversely, overexpression of TREM2 could downregulate the expressions of IL-6, IL-1β and TNF-α. Overexpression of TREM2 could also shift the microglial phenotype by increasing M2 microglia but decreasing M1 microglia in LPS-induced microglia. These indicated that upregulation of TREM2 could inhibit neuroinflammation and improve cognitive function in AD mice.

FoxO3a, a transcription factor involved in the forkhead box O family, plays an important role in brain homeostasis and development in the CNS. The function of FoxO3a was governed by PTM which controls its subcellular localization and transcriptional activity [40]. One of the main upstream regulators of FoxO3a was PI3K/AKT pathway. When AKT is activated it phosphorylates FoxO3a to become p-FoxO3a, and the latter would translocate from the nucleus to the cytoplasm. p-FoxO3a is unable to regulate its targeting genes, such as autophagy-associated genes [27]. TREM2 upregulation inhibits inflammation and alleviates neuronal injury and oxidation through PI3K/AKT pathway activation [28]. Thus, FoxO3a might be a downstream molecule of TREM2 and behind the PI3K/AKT pathway. Recent study demonstrated that autophagy was suppressed by downregulating autophagy-related genes (ATG) through the PI3K-AKT-FoxO3a signaling pathway in activated microglia [41]. According to these studies, FoxO3a might take an important role in the inflammatory response. In our study, the data showed that TREM2 deficiency significantly downregulated the levels of p-PI3K, p-AKT, and p-FoxO3a, thus increased the expressions of IL-1β, TNF-α, IL-6. Our results suggested that TREM2 negatively associated with the levels of pro-inflammatory cytokines via PI3K/AKT/FoxO3a signaling. Meanwhile, overexpression of TREM2 in microglia significantly upregulated the levels of p-PI3K, p-AKT, and p-FoxO3a followed by the decrease of the expressions of IL-1β, TNF-α, IL-6. Moreover, TREM2 inhibits inflammatory cytokine levels via PI3K/AKT/FoxO3a signaling pathway and LY294002, a PI3K inhibitor, can reverse TREM2-mediated anti-inflammatory effects by blocking the PI3K-FoxO3a axis. Along these lines, we believed that TREM2 attenuated inflammation via the activation of PI3K/AKT/FoxO3a signaling pathway in 5xFAD mice.

Microglia can be divided into M0 “homostatic function molecule’’, M1 microglia “pro-inflammation effects’’, and M2 microglia “anti-inflammatory effects” [42]. Inflammation is manipulated by the coactions of M1 and M2 microglia in CNS. Single-cell RNA sequencing showed that TREM2 could promote M2 microglia activation, suggesting TREM2 could attenuate inflammatory response through modulating the microglia polarization [9]. However, the mechanism is still unknown. Here, we hypothesized that TREM2 may modulate microglia polarization by regulating transcriptional activity of FoxO3a. Interestingly, we found that overexpression of TREM2 increased M2 phenotype microglia in LPS-treated microglia through inhibited FoxO3a transcriptional activity, and this can be reversed by LY294002. Based on our data, we found that TREM2 regulated the microglia phenotype between M1 and M2 through FoxO3a subcellular localization via PI3K/AKT signaling pathway.

In this study, there was still something needed to be improved. First, although TREM2 possess multiple beneficial properties in AD pathology, including the increase of phagocytosis, anti-apoptosis, inhibition of oxidative stress, but here we only focus on inflammatory response. So we cannot exclude that TREM2 may exert its protective effects through other way, such as inactivation of NF-κB or activation of mTOR pathway [33, 34]. Second, we downregulated TREM2 in the hippocampus by injecting CRISPR/Cas9-AAV targeting at TREM2, and that can’t achieve totally to knock-out TREM2 gene. Further studies are needed to target the TREM2-induced other related effects, such as the relationship between Foxo3a and microglia polarization.

## CONCLUSIONS

The results from our research indicated TREM2 receptor could modulate the inflammatory response through PI3K/AKT/FoxO3a signaling pathway in AD mice (summed in Figure 8). TREM2 could also improve the learning and memory deficits in 5xFAD mice via the activation of the same signaling pathway. Therefore, upregulation of TREM2 or activation of PI3K/AKT/FoxO3a pathway may serve as a potential therapeutic target in AD treatment.

## MATERIALS AND METHODS

### Mouse model of AD

5xFAD mice (n= 64; 25–30g) and WT littermates (C57BL/6J mice, n=16; 25-30g) were purchased from Jackson Laboratory (MMRRC) and housed under standard condition. Six to seven mice were bred in cages (divided by sex), under the condition of 12h light/dark cycle with free access to food and water. Only 6-7 month old 5xFAD or C57BL/6J male mice were used in this study. All of the protocols were approved by the Southern Medical University Animal Ethics Committee.

### Experiment design

**Figure 9:**
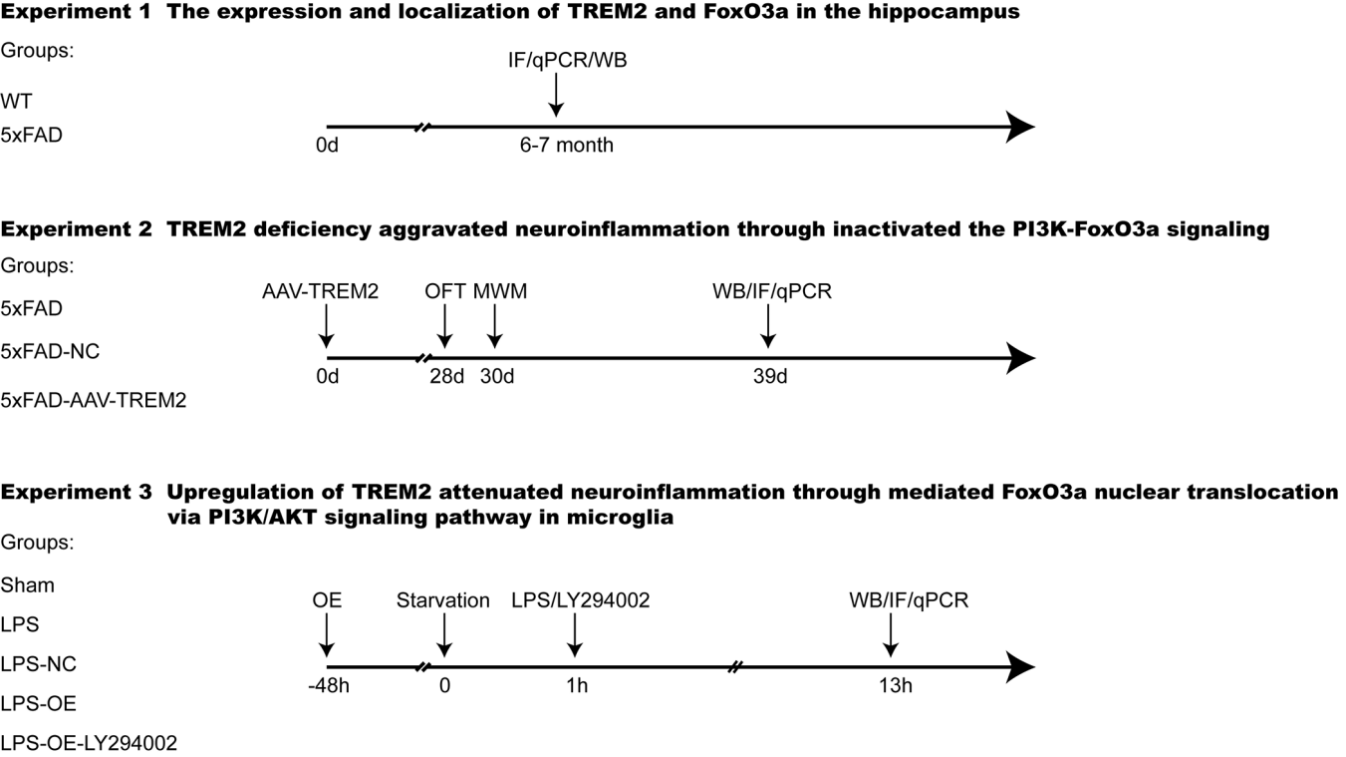
**Experimental design and groups.** TREM2, Triggering receptor expressed on myeloid cells 2; 5xFAD group was injected with PBS; 5xFAD-NC group was injected with scramble sgRNA (CRISPR/Cas9 on AAV); 5xFAD-AAV-TREM2 group was injected with TREM2 specific sgRNA (CRISPR/Cas9 on AAV). Sham, treated with PBS only; LPS, treated with lipopolysaccharide; LPS+NC, treated with LPS and negative plasmid; LPS+OE, treated with LPS and TREM2 plasmid; NfL, Neurofilament light chain; IF, immunofluorescence; LY294002, PI3K inhibitor; qPCR, Real-time PCR; WB, Western blot.

Experiments were designed as Figure 9.

### Experiment 1

To evaluate the typical pathology of 5xFAD mouse model at 6-7 months old, many AD related factors were selected for measurement. The amyloid plaques (Aβ) in hippocampus, the proteins/mRNA in the whole brain, and the serum NfL, a marker of AD were measured by Thioflavine S-straining, qPCR, and the single molecule array, respectively. We also investigated the expression and colocalization of TREM2 and FoxO3a by western blot and immunofluorescence.

### Experiment 2

To examine the effects of TREM2 on the PI3K/AKT/FoxO3a signaling pathway, downregulation of TREM2 were performed by injecting AAV into the DG area. The 5xFAD mice(n=52) were randomly divided into three groups: 5xFAD (injection of PBS; n=16), 5xFAD-AAV-NC (injection of Scramble sgRNA CRISPR/Cas9 All-in-One AAV; n=16), 5xFAD-AAV-TREM2 (injection of TREM2 sgRNA CRISPR/Cas9 All-in-One AAV; n=16). 4 mice were used to test the AAV-induced efficiency by Western blot and qPCR. Behavioral tests were performed at 28 days after AAV injection by OFT and MWM. Staining Thio S was used to assess the amyloid plaques in hippocampus at 3 days after behavior test. The PI3K/AKT/FoxO3a signaling and inflammatory response were investigated by western blot and qPCR. The expression of FoxO3a was also detected by immunofluorescence.

### Experiment 3

To further investigate TREM2-induced anti-inflammation via PI3K/AKT/FoxO3a signaling, overexpression plasmid of TREM2 and LY294002 (PI3K inhibitor) were used in BV2 cells. Cells were divided into five groups: Sham, LPS, LPS+NC (LPS+Negative vector), LPS+OE (LPS+TREM2/vector), LPS+OE+LY294002 (LPS+TREM2/vector+LY294002). Western blot and qPCR were carried out for the efficiency of transfection at 48-72 hr after plasmid transfection. Then, the PI3K/AKT/FoxO3a pathway and inflammatory response were evaluated by western blot and qPCR. Moreover, the microglia phenotype and localization of FoxO3a were detected by immunofluorescence to demonstrate that FoxO3a is involved in TREM2-regulating microglia phenotype switch.

### Cell culture and transfection

To further investigate the effects TREM2 on the PI3K/AKT/FoxO3a pathway and inflammatory response in vitro, BV2 cells (ATCC, USA) were transfected with TREM2/pEZ-M90 expression plasmid or pEZ-M90 control plasmid(vector with eGFP green fluorescence) mixed with Lipofectamin 2000 (Life Technologies, USA) at the ratio of 1ug:2 μL. To examine the transfection efficiency, these two plasmids were also transfected into 293T cells (ATCC, USA). BV2 and 293T cells were seeded in 24 wells plate at a density of 2 x 10^5^ cell/mL in Dulbecco’s Modified Eagle Medium (DMEM) containing 4.5 g/L D-glucose (Gibco, Thermo Fisher Scientific, USA) supplemented with 10% fetal bovine serum (Gibco, Lifetechnology, USA). After the plasmid mixture was added to the cells in 24-well plates, the transfected cells were firstly incubated under 5% CO2 at 37° C for 6 hr with the medium containing no FBS. Then the medium was changed to DMED containing 10%FBS and continued to incubated for 24-72 hr under the same condition. The efficiency of transfection (in 293T cell) was visualization under microscope (Olympus). For the transfected BV2 cell, the medium was replaced with non-FBS DMEM before cell harvest. The interested proteins were quantitated by western blot and/or qPCR at 24-72 hr after transfection (OE group). BV2 cells were further treated with LPS (500 ng/mL) or PBS for 12 hr after transfection (OE+LPS). In another group, BV2 cells were pretreated with LY294002 (10 μM) for 1 hr followed by adding LPS for 12 hr after transfection (OE+ LY294002+LPS).

### NFL assay

Neurofilament light chain (NfL) was detected according to previously described [30]. Briefly, blood was collected from 5xFAD and WT group mice. The serum was stored for 1 hr at 37° C to await coagulation. Then, the serum was centrifuged at 10000 x g for 5 minutes at 4° C and stored at -80° C. Blood samples were detected using single molecule array (Simoa) with a NfL kit.

### Stereotaxic microinjection

Stereotaxic microinjection was performed as previous description [43]. Briefly, the mice were anesthetized with pentobarbital sodium (50 mg/kg) and placed in a frame which was fixed the brain of the mouse. A small incision was made and a hole was drilled in the left and right of the bregma (2.0 mm lateral of the bregma). The TREM2 CRISPR all-in-one AAV (Applied Biological Materials Inc., Canada, sgRNA1: GCAAGGCCTGGTGTCGGCAG, sgRNA2: GTGATGGTGACGGTTCCAGC, sgRNA 3: ACGCGGGCCTCTACCAGTGT) or scramble CRISPR AAV (Applied Biological Materials Inc., Canada, sgRNA: GCACTCACATCGCTACATCA) were bilaterally injected into DG (AP=-2.0mm, ML=1.2mm, DV=2mm, 0.5μl, 0.5μl min-1) according the manufacturer's instructions. The needle was left in for 5 min after AAV injected and then withdrawn slowly. After the incision, these mice were closed with sutures and placed back to the original cage. The behavioral tests were conducted after twenty-eight days according to the specific property of AAV. Then, the bilateral hippocampus was taken by sacrificing the mice to evaluate the efficiency of the TREM2 knockdown by RT-PCR and western blot.

### Immunofluorescence

The immunofluorescence was performed by previously description [44]. Briefly, the mice were anesthesia with 0.75% pentobarbital sodium, and perfused with saline solution and followed with 4% paraformaldehyde. The brain was removed and then post-fixed in 4% paraformaldehyde at 4° C for 24 hr. Afterward, the brain was dehydrated in 30% sucrose solution until the brain sinks to the bottom. Samples were embedded in optimal cutting temperature compound (OCT) at -22° C followed by cutting into 25-μm-thick sections using a cryostat (CM1950; Leica, Germany). The redundant sections were collected and frozen at -80° C until used. Cells were washed four times with PBS and fixed with 4% paraformaldehyde. The sections and/or cells were blocked by 5% BSA containing 1% triton X-100 at 37° C for 2 hr and followed by incubated overnight at 4° C, then incubated with various primary antibodies: anti-Iba-1 (1:300, Millipore, NP001614), anti-TREM2 (1:1000, R&D System, AF1729), anti-FoxO3a (1:1000, Thermo Fisher Scientific, MA5-14932), anti-CD206 (1:500, R&D System, AF2535), anti-CD32 (1:500,R&D System, AF21460), anti-p-FoxO3a (1:500, Boster, BM4401). After washing four times with PBS, the corresponding secondary antibodies (1:5000) were incubated at room temperature for 1.5 hr and next mounted onto the slide with DAPI.

Thioflavine S (Thio S, Sigma, USA) were diluted with 50% ethanol. Brain sections were strained with 1% Thio S for 5 min, then washing the sections with 70% ethanol for 4 times followed by 3 times wash with PBS. The samples were visualized using confocal microscopy (Nikon; Japan) and harvested a photograph to analysis the interested area. The results were analyzed in four to five different fields in the DG area per mouse by image J. The data were showed with cells/field.

### Western blot

To examine the protein levels of PI3K/AKT/FoxO3a pathway and inflammatory cytokines conducted by TREM2, all of the samples were prepared for western blot. Western blot was performed as described previously [30]. Briefly, hippocampi or microglial cells were lysed in RIPA buffer (including phosphatase inhibitor, cocktail protease inhibitors) and next collected the supernatant after that the samples were centrifuged at 12,000 x g for 25 min at 4° C. The concentration of the total proteins was detected by BCA (Thermo Fisher, USA) according to manufacturer’s instruction. All of proteins were boiled with working SDS loading buffer at 100° C for 15 min. Total protein (25 μg) of each sample was loaded onto 4-12% gel and then proteins were transferred onto 0.45 μM polyvinylidene difluoride (PVDF) membranes (Milipore, USA). Next, the membranes were blocked using 5% milk at room temperature for 1.5 hr. The interested proteins were probed by specific primary antibodies overnight at 4° C (anti-GAPDH 1:3000, Cell Signaling Technology); anti-actin (1:1000, Cell Signaling Technology); anti-TRME2 (1:3000, R&D System); anti-FoxO3a (1:1000, Thermo Fisher Scientific); anti-phospho-FoxO3a (1:1000, BOSTER); anti-PI3K (1:1000,Cell Signaling Technology); anti-phospho-PI3K (1:1000, Cell Signaling Technology); anti-AKT(1:1000, BOSTER); anti-phospho-AKT (1:500, AB clonal); anti-IL-1β (1:1000, BOSTER); anti-TNF-α (1:1000, BOSTER). Then, blots were incubated with homologous secondary antibodies (1:5000, BOSTER) at room temperature for 1.5 hr. The bands were visualized with ECL regent (FdBio Science) and exposed to BioRad image system. The intensity of bands was analyzed by image J software.

### Quantitative real-time PCR

To detect the mRNA levels of TREM2, FoxO3a and inflammatory cytokines (eg IL-1β, IL-6, TNF-α), the total RNA was extracted from hippocampus tissue and cells using Hi-pure universal RNA kit (Magen) according to the manufacturer's protocol. The concentration of mRNA was determined by Nanodrop 2000 (Thermo Fisher Scientific). The cDNA was synthesized using iScript cDNA Synthesis kit (Bio-Rad), and the reaction of qRT-PCR was performed by GoScript^TM^qPCR Marster Mix (Promega, USA). Targeting genes were amplified by the following primers: TREM2 (sense primer: 5′-CTG GAA CCG TCA CCA TCA CT-3′, antisense primer: 5′-CAC CCT CGA AAC TCG ATG AC-3′); FoxO3a (sense primer: 5′-GAG TGA CTC CAG CAG CCT TG-3′, antisense primer: 5′-ATT CCA AGC TCC CAT TGA AC-3′); TNF-α (sense primer: 5′-TCA CTG GAG CCT CGA ATG TC-3′, antisense primer: 5′-TCT GTG AGG AAG GCT GTG CA-3′); IL-1β (sense primer:5′-TGT GTA ATG AAA GAC GGC ACA C-3′, antisense primer: 5′-CTT GTG AGG TGC TGA TGT ACC A-3′); IL-6 (sense primer: 5′-CCA CGG CCT TCC CTA CTT C-3′, antisense primer: 5′-TTG GGA GTG GTA TCC TCT GTG A -3′); GAPDH (sense primer: 5′-AGT GTT TCC TCG TCC CGT AGA-3′, antisense primer: 5′- TTG CCG TGA GTG GAG TCA TAC-3′); The gene of GAPDH was considered as housekeeping gene and the expression of the interested genes were all normalized to GAPDH. The data was analyzed using the comparative CT method.

### Edu assay

To examine the effects of TREM2 on proliferation of BV2, Edu assay kit (BeyoClick TM, China) was purchased. BV2 microglia were seeded in 24-well plates (1 x 10^5^ cells/well), and then treated with PBS, negative vector or TREM2/vector (1 μg/well) for 12 hr and then added the Edu working solution into culture medium. After that, microglial cells were fixed with 4% PFA for 15 min at room temperature and then the nucleus were labeled with DAPI for 10 min. The results were visualized by confocal microscope at λ450 nm.

### Morris water maze

The spatial memory was evaluated by Morris water maze (MWM) as described previously [45]. MWM was assessed in a circular pool (diameter 120 cm), which is made of polypropylene, filled with opacified water and maintained at 23° C (height 30 cm) and divided into four quadrants. A 10-cm circular opaque platform was placed 1 cm below the water surface. There the tested mice were released facing the wall from four quadrants in one day and free swim within 90 seconds. If the mice fail to find the hidden platform, the mice were guided into the hidden platform and stay 15 seconds and remove otherwise. The spatial probe trial was performed after 24 h from the last training session, remove the platform under the water level and release the mice from the contrary quadrant placing the hidden platform, record the percent time that the mice stayed in the hidden platform. After that, a cued test was performed by placing a submerged platform with a flag above the water level at the opposite side. One trial per mouse was implemented and the time to the visible platform was recorded.

### Open field test

The rectangular chamber was employed in this test, which was made of gray polyvinyl chloride, to evaluate the muscular motor activity. A video camera was used to record the activity in the chamber. The mice were gently released into the center of the apparatus and to freely explore the new area for 5 min. The data of each mouse was recorded and stored, total distance and center times were analyzed using Etho Vision 7.0 software.

### Statistical analyses

All the statistical results were expressed by mean ± SEM (standard error of the mean). Statistical analysis was determined by GraphPad Prism 7.0 software. Quantification of confocal image or gray density was performed using Image J. The investigators were blinded to the genotype for behavioral tests and all the quantifications. Comparisons between two groups were analyzed by unpaired t test. Comparisons of multiple groups were evaluated using one-way ANOVA followed by Tukey's post hoc test. Data from MWM were performed by two-way repeated-measures ANOVA followed by Tukey's post hoc test. The differences between the two groups were performed using the unpaired T-test. The significant difference was accepted if p ≤ 0.05.

### Ethics approval

This study was approved by the Animal Ethics Committee of Southern Medical University in accordance with the National Institutes of Health guidelines for the care and use of experimental animals.

## References

[1] Holtzman DM, Morris JC, Goate AM. Alzheimer’s disease: the challenge of the second century. Sci Transl Med. 2011; 3:77sr1. 10.1126/scitranslmed.3002369 21471435 PMC3130546

[2] Huang Y, Mucke L. Alzheimer mechanisms and therapeutic strategies. Cell. 2012; 148:1204–22. 10.1016/j.cell.2012.02.040 22424230 PMC3319071

[3] Prince M, Wimo A, Guerchet M, Ali GC, Wu YT, Prina M. The global impact of dementia. An analysis of prevalence, incidence, cost and trends. London: Published by Alzheimer’s Disease International (ADI) 2015.

[4] Crotti A, Ransohoff RM. Microglial physiology and pathophysiology: insights from genome-wide transcriptional profiling. Immunity. 2016; 44:505–15. 10.1016/j.immuni.2016.02.013 26982357

[5] Hansen DV, Hanson JE, Sheng M. Microglia in Alzheimer’s disease. J Cell Biol. 2018; 217:459–72. 10.1083/jcb.201709069 29196460 PMC5800817

[6] Perry VH, Holmes C. Microglial priming in neurodegenerative disease. Nat Rev Neurol. 2014; 10:217–24. 10.1038/nrneurol.2014.38 24638131

[7] Guerreiro R, Wojtas A, Bras J, Carrasquillo M, Rogaeva E, Majounie E, Cruchaga C, Sassi C, Kauwe JS, Younkin S, Hazrati L, Collinge J, Pocock J, et al, and Alzheimer Genetic Analysis Group. TREM2 variants in Alzheimer’s disease. N Engl J Med. 2013; 368:117–27. 10.1056/NEJMoa1211851 23150934 PMC3631573

[8] Jonsson T, Stefansson H, Steinberg S, Jonsdottir I, Jonsson PV, Snaedal J, Bjornsson S, Huttenlocher J, Levey AI, Lah JJ, Rujescu D, Hampel H, Giegling I, et al. Variant of TREM2 associated with the risk of Alzheimer’s disease. N Engl J Med. 2013; 368:107–16. 10.1056/NEJMoa1211103 23150908 PMC3677583

[9] Mazaheri F, Snaidero N, Kleinberger G, Madore C, Daria A, Werner G, Krasemann S, Capell A, Trümbach D, Wurst W, Brunner B, Bultmann S, Tahirovic S, et al. TREM2 deficiency impairs chemotaxis and microglial responses to neuronal injury. EMBO Rep. 2017; 18:1186–98. 10.15252/embr.201743922 28483841 PMC5494532

[10] Ulland TK, Song WM, Huang SC, Ulrich JD, Sergushichev A, Beatty WL, Loboda AA, Zhou Y, Cairns NJ, Kambal A, Loginicheva E, Gilfillan S, Cella M, et al. TREM2 maintains microglial metabolic fitness in Alzheimer’s disease. Cell. 2017; 170:649–63.e13. 10.1016/j.cell.2017.07.023 28802038 PMC5573224

[11] Cannon JP, O’Driscoll M, Litman GW. Specific lipid recognition is a general feature of CD300 and TREM molecules. Immunogenetics. 2012; 64:39–47. 10.1007/s00251-011-0562-4 21800138

[12] Yeh FL, Wang Y, Tom I, Gonzalez LC, Sheng M. TREM2 binds to apolipoproteins, including APOE and CLU/APOJ, and thereby facilitates uptake of amyloid-beta by microglia. Neuron. 2016; 91:328–40. 10.1016/j.neuron.2016.06.015 27477018

[13] Turnbull IR, Gilfillan S, Cella M, Aoshi T, Miller M, Piccio L, Hernandez M, Colonna M. Cutting edge: TREM-2 attenuates macrophage activation. J Immunol. 2006; 177:3520–24. 10.4049/jimmunol.177.6.3520 16951310

[14] Peng Q, Malhotra S, Torchia JA, Kerr WG, Coggeshall KM, Humphrey MB. TREM2- and DAP12-dependent activation of PI3K requires DAP10 and is inhibited by SHIP1. Sci Signal. 2010; 3:ra38. 10.1126/scisignal.2000500 20484116 PMC2900152

[15] Wang Y, Cella M, Mallinson K, Ulrich JD, Young KL, Robinette ML, Gilfillan S, Krishnan GM, Sudhakar S, Zinselmeyer BH, Holtzman DM, Cirrito JR, Colonna M. TREM2 lipid sensing sustains the microglial response in an Alzheimer’s disease model. Cell. 2015; 160:1061–71. 10.1016/j.cell.2015.01.049 25728668 PMC4477963

[16] Griciuc A, Patel S, Federico AN, Choi SH, Innes BJ, Oram MK, Cereghetti G, McGinty D, Anselmo A, Sadreyev RI, Hickman SE, El Khoury J, Colonna M, Tanzi RE. TREM2 acts downstream of CD33 in modulating microglial pathology in Alzheimer’s disease. Neuron. 2019; 103:820–35.e7. 10.1016/j.neuron.2019.06.010 31301936 PMC6728215

[17] Song W, Hooli B, Mullin K, Jin SC, Cella M, Ulland TK, Wang Y, Tanzi RE, Colonna M. Alzheimer’s disease-associated TREM2 variants exhibit either decreased or increased ligand-dependent activation. Alzheimers Dement. 2017; 13:381–87. 10.1016/j.jalz.2016.07.004 27520774 PMC5299056

[18] Lessard CB, Malnik SL, Zhou Y, Ladd TB, Cruz PE, Ran Y, Mahan TE, Chakrabaty P, Holtzman DM, Ulrich JD, Colonna M, Golde TE. High-affinity interactions and signal transduction between Aβ oligomers and TREM2. EMBO Mol Med. 2018; 10:e9027. 10.15252/emmm.201809027 30341064 PMC6220267

[19] Jiang T, Tan L, Zhu XC, Zhang QQ, Cao L, Tan MS, Gu LZ, Wang HF, Ding ZZ, Zhang YD, Yu JT. Upregulation of TREM2 ameliorates neuropathology and rescues spatial cognitive impairment in a transgenic mouse model of Alzheimer’s disease. Neuropsychopharmacology. 2014; 39:2949–62. 10.1038/npp.2014.164 25047746 PMC4229581

[20] Hamerman JA, Tchao NK, Lowell CA, Lanier LL. Enhanced toll-like receptor responses in the absence of signaling adaptor DAP12. Nat Immunol. 2005; 6:579–86. 10.1038/ni1204 15895090 PMC1282462

[21] Willcox BJ, Donlon TA, He Q, Chen R, Grove JS, Yano K, Masaki KH, Willcox DC, Rodriguez B, Curb JD. FOXO3A genotype is strongly associated with human longevity. Proc Natl Acad Sci USA. 2008; 105:13987–92. 10.1073/pnas.0801030105 18765803 PMC2544566

[22] Daitoku H, Sakamaki J, Fukamizu A. Regulation of FoxO transcription factors by acetylation and protein-protein interactions. Biochim Biophys Acta. 2011; 1813:1954–60. 10.1016/j.bbamcr.2011.03.001 21396404

[23] Zhang X, Wang L, Peng L, Tian X, Qiu X, Cao H, Yang Q, Liao R, Yan F. Dihydromyricetin protects HUVECs of oxidative damage induced by sodium nitroprusside through activating PI3K/Akt/FoxO3a signalling pathway. J Cell Mol Med. 2019; 23:4829–38. 10.1111/jcmm.14406 31111658 PMC6584490

[24] Qin W, Zhao W, Ho L, Wang J, Walsh K, Gandy S, Pasinetti GM. Regulation of forkhead transcription factor FoxO3a contributes to calorie restriction-induced prevention of Alzheimer’s disease-type amyloid neuropathology and spatial memory deterioration. Ann N Y Acad Sci. 2008; 1147:335–47. 10.1196/annals.1427.024 19076455 PMC2605640

[25] Shang YC, Chong ZZ, Hou J, Maiese K. The forkhead transcription factor FOXO3a controls microglial inflammatory activation and eventual apoptotic injury through caspase 3. Curr Neurovasc Res. 2009; 6:20–31. 10.2174/156720209787466064 19355923 PMC2668140

[26] Netea-Maier RT, Plantinga TS, van de Veerdonk FL, Smit JW, Netea MG. Modulation of inflammation by autophagy: consequences for human disease. Autophagy. 2016; 12:245–60. 10.1080/15548627.2015.1071759 26222012 PMC4836004

[27] Cao Y, Chen J, Ren G, Zhang Y, Tan X, Yang L. Punicalagin prevents inflammation in LPS-induced RAW264.7 macrophages by inhibiting FoxO3a/autophagy signaling pathway. Nutrients. 2019; 11:2794. 10.3390/nu11112794 31731808 PMC6893462

[28] Liu AH, Chu M, Wang YP. Up-regulation of Trem2 inhibits hippocampal neuronal apoptosis and alleviates oxidative stress in epilepsy via the PI3K/Akt pathway in mice. Neurosci Bull. 2019; 35:471–85. 10.1007/s12264-018-0324-5 30684126 PMC6527642

[29] Yoon HE, Kim SJ, Kim SJ, Chung S, Shin SJ. Tempol attenuates renal fibrosis in mice with unilateral ureteral obstruction: the role of PI3K-Akt-FoxO3a signaling. J Korean Med Sci. 2014; 29:230–37. 10.3346/jkms.2014.29.2.230 24550650 PMC3924002

[30] Besse M, Belz M, Folsche T, Vogelgsang J, Methfessel I, Steinacker P, Otto M, Wiltfang J, Zilles D. Serum neurofilament light chain (NFL) remains unchanged during electroconvulsive therapy. World J Biol Psychiatry. 2020; 21:148–54. 10.1080/15622975.2019.1702717 31818180

[31] Heneka MT, Golenbock DT, Latz E. Innate immunity in Alzheimer’s disease. Nat Immunol. 2015; 16:229–36. 10.1038/ni.3102 25689443

[32] Freeman LC, Ting JP. The pathogenic role of the inflammasome in neurodegenerative diseases. J Neurochem. 2016 (Suppl 1); 136:29–38. 10.1111/jnc.13217 26119245

[33] Zhong L, Chen XF, Wang T, Wang Z, Liao C, Wang Z, Huang R, Wang D, Li X, Wu L, Jia L, Zheng H, Painter M, et al. Soluble TREM2 induces inflammatory responses and enhances microglial survival. J Exp Med. 2017; 214:597–607. 10.1084/jem.20160844 28209725 PMC5339672

[34] Zusso M, Lunardi V, Franceschini D, Pagetta A, Lo R, Stifani S, Frigo AC, Giusti P, Moro S. Ciprofloxacin and levofloxacin attenuate microglia inflammatory response via TLR4/NF-kB pathway. J Neuroinflammation. 2019; 16:148. 10.1186/s12974-019-1538-9 31319868 PMC6637517

[35] Boza-Serrano A, Ruiz R, Sanchez-Varo R, García-Revilla J, Yang Y, Jimenez-Ferrer I, Paulus A, Wennström M, Vilalta A, Allendorf D, Davila JC, Stegmayr J, Jiménez S, et al. Galectin-3, a novel endogenous TREM2 ligand, detrimentally regulates inflammatory response in Alzheimer’s disease. Acta Neuropathol. 2019; 138:251–73. 10.1007/s00401-019-02013-z 31006066 PMC6660511

[36] Matarin M, Salih DA, Yasvoina M, Cummings DM, Guelfi S, Liu W, Nahaboo Solim MA, Moens TG, Paublete RM, Ali SS, Perona M, Desai R, Smith KJ, et al. A genome-wide gene-expression analysis and database in transgenic mice during development of amyloid or tau pathology. Cell Rep. 2015; 10:633–44. 10.1016/j.celrep.2014.12.041 25620700

[37] Srinivasan K, Friedman BA, Larson JL, Lauffer BE, Goldstein LD, Appling LL, Borneo J, Poon C, Ho T, Cai F, Steiner P, van der Brug MP, Modrusan Z, et al. Untangling the brain’s neuroinflammatory and neurodegenerative transcriptional responses. Nat Commun. 2016; 7:11295. 10.1038/ncomms11295 27097852 PMC4844685

[38] Jay TR, von Saucken VE, Landreth GE. TREM2 in neurodegenerative diseases. Mol Neurodegener. 2017; 12:56. 10.1186/s13024-017-0197-5 28768545 PMC5541421

[39] Kim SM, Mun BR, Lee SJ, Joh Y, Lee HY, Ji KY, Choi HR, Lee EH, Kim EM, Jang JH, Song HW, Mook-Jung I, Choi WS, Kang HS. TREM2 promotes Aβ phagocytosis by upregulating C/EBPα-dependent CD36 expression in microglia. Sci Rep. 2017; 7:11118. 10.1038/s41598-017-11634-x 28894284 PMC5593901

[40] Morris BJ, Willcox DC, Donlon TA, Willcox BJ. FOXO3: a major gene for human longevity—a mini-review. Gerontology. 2015; 61:515–25. 10.1159/000375235 25832544 PMC5403515

[41] Lee JW, Nam H, Kim LE, Jeon Y, Min H, Ha S, Lee Y, Kim SY, Lee SJ, Kim EK, Yu SW. TLR4 (toll-like receptor 4) activation suppresses autophagy through inhibition of FOXO3 and impairs phagocytic capacity of microglia. Autophagy. 2019; 15:753–70. 10.1080/15548627.2018.1556946 30523761 PMC6526818

[42] Cherry JD, Olschowka JA, O’Banion MK. Neuroinflammation and M2 microglia: the good, the bad, and the inflamed. J Neuroinflammation. 2014; 11:98. 10.1186/1742-2094-11-98 24889886 PMC4060849

[43] Guo N, Soden ME, Herber C, Kim MT, Besnard A, Lin P, Ma X, Cepko CL, Zweifel LS, Sahay A. Dentate granule cell recruitment of feedforward inhibition governs engram maintenance and remote memory generalization. Nat Med. 2018; 24:438–49. 10.1038/nm.4491 29529016 PMC5893385

[44] Xie Z, Huang L, Enkhjargal B, Reis C, Wan W, Tang J, Cheng Y, Zhang JH. Intranasal administration of recombinant netrin-1 attenuates neuronal apoptosis by activating DCC/APPL-1/AKT signaling pathway after subarachnoid hemorrhage in rats. Neuropharmacology. 2017; 119:123–33. 10.1016/j.neuropharm.2017.03.025 28347836 PMC5490977

[45] Vorhees CV, Williams MT. Morris water maze: procedures for assessing spatial and related forms of learning and memory. Nat Protoc. 2006; 1:848–58. 10.1038/nprot.2006.116 17406317 PMC2895266

